# Public health round-up

**DOI:** 10.2471/BLT.17.010817

**Published:** 2017-08-01

**Climate change and health**

WHO launched the second round of its *Climate and health country profiles* last month. The profiles provide updated scientific evidence on the health risks in countries related to global warming. These boys are fishing in the city of Korhoga in Côte d'Ivoire, where evidence on water shortages and extreme weather events will inform strategies for community resilience to climate change. http://www.who.int/globalchange/resources/countries

**Figure:**
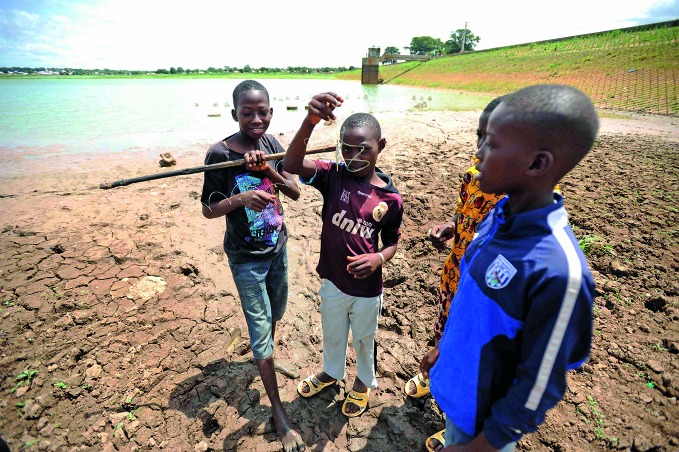
WHO/TDR /Andy Craggs

## Yemen faces world’s worst cholera outbreak

Twenty ambulances, 100 cholera kits and other equipment, and 128 000 bags of intravenous fluids were among a 403-tonne WHO shipment that arrived in Yemen last month.

But delivering medical supplies to vulnerable people across Yemen is a major logistical challenge in this conflict-affected country with its damaged port infrastructures. 

In June, outgoing WHO Director-General Margaret Chan and Executive Director Anthony Lake of the United Nations Children’s Fund (UNICEF) issued a statement highlighting the rapidly spreading cholera outbreak with more than 200 000 suspected cases, increasing at an average of 5000 a day.

“We are now facing the worst cholera outbreak in the world. In just two months, cholera has spread to almost every governorate of this war-torn country. Already more than 1300 people have died – one quarter of them children – and the death toll is expected to rise,” they said.

The agencies have been working around the clock to detect and track the spread of disease. Rapid response teams have been going house-to-house to tell families how they can protect themselves by cleaning their hands and by boiling and storing drinking water.

“We call on authorities in Yemen to strengthen their internal efforts to stop the outbreak from spreading further. This deadly cholera outbreak is the direct consequence of two years of heavy conflict,” Chan and Lake said.

As a result of collapsing health, water and sanitation systems about 14.5 million people have been cut off from regular access to clean water and sanitation leading to the rapid spread of the disease. 

About 30 000 local health workers, who are working to end the outbreak, have not been paid their salaries for nearly 10 months.

“We urge all authorities inside the country to pay these salaries and, above all, we call on all parties to end this devastating conflict,” Chan and Lake said.

http://www.who.int/hac/crises/yem/

## Ebola outbreak ends in the Democratic Republic of the Congo

WHO declared the end of a recent outbreak of Ebola virus disease in the Democratic Republic of the Congo last month.

The announcement came 42 days – two 21-day incubation cycles of the virus – after the last confirmed Ebola patient tested negative for the disease for the second time.

WHO said that enhanced surveillance for possible further Ebola cases will continue, while preparedness and readiness for further Ebola outbreaks will continue to be strengthened in the central African country.

"With the end of this epidemic, DRC has once again proved to the world that we can control the very deadly Ebola virus if we respond early in a coordinated and efficient way,” said Dr Tedros Adhanom Ghebreyesus, WHO Director-General, who took office last month.

Four people died and four others survived the disease. Five of these eight cases were laboratory confirmed. A total of 583 people, who had been in contact with these eight people, were registered and closely monitored, but none developed signs or symptoms of the disease.

The Ministry of Public Health in the Democratic Republic of the Congo notified WHO of a cluster of illnesses and deaths with haemorrhagic symptoms in people living in the Likati Health Zone in May, from whom one blood sample tested positive for Ebola.

Likati is a remote area on the border of the Central African Republic. Cases of the disease were reported in four health districts. It was the eighth Ebola outbreak in the Democratic Republic of the Congo since the virus was first identified in the country in 1976.

The outbreak was quelled thanks to the timely alert by local authorities of suspected cases, the prompt testing of blood samples due to strengthened national laboratory capacity and the early announcement of the outbreak by the Government of the Democratic Republic of the Congo.

The WHO Health Emergencies Programme helped coordinate outbreak control efforts on the ground and set up incident management system within 24 hours of the outbreak being announced.

http://www.who.int/emergencies/ebola-DRC-2017

**Cover photo**

One of Ethiopia’s 38 000 female health extension workers treks along a dirt track in the Tigray region carrying a box of medicines on her back. Ethiopia’s community health workers play a crucial role in addressing the health needs of the country’s population.

**Figure:**
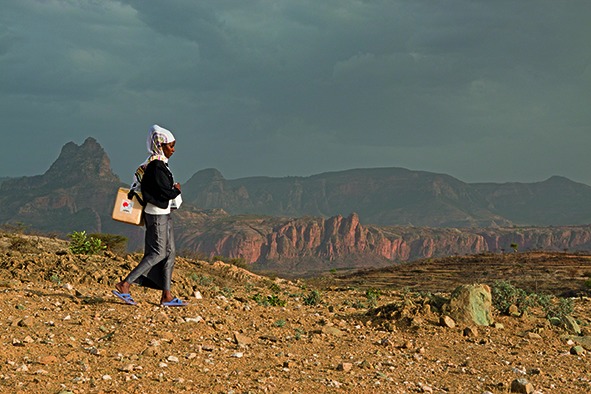
Global Fund/Petterik Wiggers

## More prevention, new drugs needed for gonorrhoea

Data from 77 countries released last month show that antibiotic resistance is making gonorrhoea – a common sexually-transmitted infection – much harder, and sometimes impossible, to treat.

WHO reports widespread resistance to older and cheaper antibiotics. Some countries – particularly high-income ones, where surveillance for communicable diseases is strong – are finding cases of the infection that are untreatable by all known antibiotics.

Each year, an estimated 78 million people are infected with gonorrhoea. Gonorrhoea can infect the genitals, rectum and throat. The complications in women can be particularly serious, including pelvic inflammatory disease, ectopic pregnancy and infertility.

Decreasing condom use, higher numbers of sexual partners exposed through urbanization and travel, poor infection detection rates, and inadequate or failed treatment all contribute to the spread of antibiotic-resistant strains.

The new data are from the WHO Global Gonococcal Antimicrobial Surveillance Programme (WHO GASP), which monitors trends in drug-resistant gonorrhoea.

Gonorrhoea can be prevented through safer sexual behaviour, especially consistent and correct condom use, while information and education can help promote such practices.

More research and development for gonorrhoea treatment are needed, as currently there are only three candidate drugs in various stages of clinical development.

Last year the Drugs for Neglected Diseases initiative and WHO launched the Global Antibiotic Research and Development Partnership, a not-for-profit research and development organization to help address the shortage of new antibiotics.

http://www.who.int/mediacentre/news/releases/2017/Antibiotic-resistant-gonorrhoea

## WHO calls for action on sunbeds

WHO calls on countries to ban or restrict the use of artificial tanning devices (sunbeds) as these expose people unnecessarily to ultraviolent radiation which causes skin cancer.

More than 40 national and provincial authorities around the world have now banned or restricted the use of sunbeds.

According to a new WHO report*,* sunbeds pose a specific risk for melanoma, independent of skin type and of sun exposure. The risk of melanoma increases with younger age of first sunbed use and with greater lifetime use of sunbeds, according to *Artificial tanning devices: public health interventions to manage sunbeds*.

Research shows that people who have used a sunbed at least once at any stage of their life have a 20% higher risk of developing melanoma than people who have never used a sunbed. Use of sunbeds before the age of 35 increases the risk of developing melanoma by 59%.

Sunbed use is estimated to be responsible for more than 450 000 non-melanoma skin cancer cases and more than 10 000 melanoma cases every year in Australia, Europe and the United States of America combined. Most sunbed users are young women.

In 2009, WHO’s International Agency for Research on Cancer classified the use of ultraviolent-emitting tanning devices as carcinogenic to humans.

Brazil and Australia have banned commercial sunbeds. Most countries that have sunbed regulations prohibit people under the age of 18 from using sunbeds. Canada, France, Ireland and the United States of America restrict sunbed operators from advertising non-cosmetic health benefits while in Italy legislative controls require sunbed operators to prohibit use by pregnant women and fair-skinned people.

http://www.who.int/uv/publications/artificial-tanning-devices/en/

## New ethics guidance for public health surveillance

WHO has issued new advice – *Guidelines on ethical issues in public health surveillance –* that policy-makers and practitioners can use to navigate the ethical issues presented by public health surveillance.

Surveillance, when conducted ethically, is the foundation for public health programmes and can provide evidence for coverage and effectiveness of many disease control interventions.

However, surveillance is not without risks for participants and sometimes poses ethical dilemmas. 

WHO recommends that surveillance systems should have a clear purpose and a plan for data collection, analysis, use and dissemination based on public health priorities. Countries should develop appropriate, effective mechanisms to ensure ethical surveillance and these data should be collected only for a legitimate public health purpose. The global community has an obligation to support countries that lack adequate resources to undertake surveillance.

**Looking ahead**

13–16 September – Global Evidence Summit, Cape Town, South Africa

18–20 October – WHO Global Conference on Noncommunicable Diseases, Montevideo, Uruguay

1–3 November – World Hepatitis Summit 2017, São Paulo, Brazil

16–17 November – Global Ministerial Conference on Tuberculosis, Moscow, Russian Federation

